# The COP9 signalosome is vital for timely repair of DNA double-strand breaks

**DOI:** 10.1093/nar/gkv270

**Published:** 2015-04-08

**Authors:** Michal Meir, Yaron Galanty, Lior Kashani, Michael Blank, Rami Khosravi, María Jesús Fernández-Ávila, Andrés Cruz-García, Ayelet Star, Lea Shochot, Yann Thomas, Lisa J. Garrett, Daniel A. Chamovitz, David M. Bodine, Thimo Kurz, Pablo Huertas, Yael Ziv, Yosef Shiloh

**Affiliations:** 1 The David and Inez Myers Laboratory for Cancer Research, Department of Human Molecular Genetics and Biochemistry, Sackler School of Medicine, George S. Wise Faculty of Life sciences, Tel Aviv University, Tel Aviv, 69978 Israel; 2 Centro Andaluz de Biología Molecular y Medicina Regenerativa (CABIMER) and Department of Genetics, University of Sevilla, Sevilla, 41092, Spain; 3 MRC Protein Phosphorylation and Ubiquitylation Unit, College of Life Sciences, University of Dundee, Dundee, DD1 5EH, Scotland, UK; 4 Genetics and Molecular Biology Branch, National Human Genome Research Institute, National Institutes of Health, Bethesda, MD, 20892, USA; 5 Department of Molecular Biology and Ecology of Plants, George S. Wise Faculty of Life sciences, Tel Aviv University, Tel Aviv, 69978, Israel

## Abstract

The DNA damage response is vigorously activated by DNA double-strand breaks (DSBs). The chief mobilizer of the DSB response is the ATM protein kinase. We discovered that the COP9 signalosome (CSN) is a crucial player in the DSB response and an ATM target. CSN is a protein complex that regulates the activity of cullin ring ubiquitin ligase (CRL) complexes by removing the ubiquitin-like protein, NEDD8, from their cullin scaffold. We find that the CSN is physically recruited to DSB sites in a neddylation-dependent manner, and is required for timely repair of DSBs, affecting the balance between the two major DSB repair pathways—nonhomologous end-joining and homologous recombination repair (HRR). The CSN is essential for the processivity of deep end-resection—the initial step in HRR. Cullin 4a (CUL4A) is recruited to DSB sites in a CSN- and neddylation-dependent manner, suggesting that CSN partners with CRL4 in this pathway. Furthermore, we found that ATM-mediated phosphorylation of CSN subunit 3 on S410 is critical for proper DSB repair, and that loss of this phosphorylation site alone is sufficient to cause a DDR deficiency phenotype in the mouse. This novel branch of the DSB response thus significantly affects genome stability.

## INTRODUCTION

The DNA damage response (DDR) constitutes a central axis in the maintenance of genome stability (1–3). The DDR is an extensive signaling network, based on a core of dedicated damage response proteins that is assisted by a multitude of other proteins, which are temporarily recruited from various pathways to serve the DDR. The DDR is activated most vigorously by DNA double-strand breaks (DSBs). The DSB response activates DNA repair mechanisms and special cell cycle checkpoints, thereby modulating numerous cellular circuits while the damage is repaired (1,3). Its early phase is marked by the recruitment of a heterogeneous group of proteins to DSB sites, collectively dubbed ‘sensors’ or ‘mediators’. These proteins coalesce into highly ordered structures, visible as nuclear foci at the break sites (4), whose activity leads to chromatin reorganization and transcription arrest at the sites of DNA damage and sets the scene for DSB repair (5). This activity is regulated by extensive protein post-translational modifications, such as phosphorylation and ubiquitylation, which occur on the sensors/mediators as well as on chromatin proteins including core histones (4–8). DSB repair is carried out under the precise regulation of several repair mechanisms, divided into two branches: classical nonhomologous end-joining (NHEJ) and its sub-pathways, and homologous recombination repair (HRR) (3). Briefly, NHEJ is based on direct ligation of DSB ends following their initial processing; it takes place throughout the cell cycle via several different mechanisms, and is considered mutagenic. HRR, on the other hand, is error-free; it is based on recombination between the damaged DNA molecule and an undamaged sister molecule, and can hence occur only at the late S or G2 phases of the cell cycle. Maintaining the balance between these two repair pathways, which can potentially compete for repair of the same lesion, is important for efficient and timely DSB repair (9).

The chief mobilizer of the DSB response is the homeostatic, multi-functional protein kinase ATM, whose activity is markedly enhanced following DSB induction (10,11). Activated ATM phosphorylates numerous effectors in the various branches of the DDR, mobilizing this intricate network in a concerted manner (10–14). ATM is a serine-threonine protein kinase with a PI3-kinase signature. It is a member of the PI3 kinase-related protein kinase (PIKK) family. This family includes, among others, the catalytic subunit of the DNA-dependent protein kinase (DNA-PKcs), which is a key player in the NHEJ branch of DSB repair and probably also in other genotoxic stress responses (15,16), and ATR, which responds primarily to stalled replication forks (17,18). The three protein kinases, which often collaborate in maintaining genome stability, preferably phosphorylate Ser or Thr residues followed by Gln (S/TQ motif).

Here, we report a novel, vital role for the COP9 signalosome (CSN) in the early phase of the DSB response, affecting the choice between DSB repair pathways. CSN is a eukaryotic, evolutionarily conserved protein complex that is located both in the nucleus and cytoplasm. It plays critical developmental roles in animals and plants by impacting many signaling pathways (19–21). Not surprisingly, loss of CSN subunits in the mouse is embryonic lethal (22,23). CSN is composed of eight subunits (CSN1–8) that act as a holoenzyme, with Zn^2+^-dependent isopeptidase activity residing in subunit 5 (CSN5) (24). This activity removes a covalently conjugated ubiquitin-like protein, NEDD8, from cullin proteins. Members of the cullin family (eight in mammals) (25) serve as scaffolds in cullin RING ubiquitin ligases (CRLs)—highly modular protein complexes that constitute the largest group of ubiquitin E3 ligases (26–28). CRLs are defined primarily by the nature of their cullin scaffold and their numerous exchanging substrate receptor modules. The continuous exchange of the substrate receptors allows CRLs to act dynamically in different contexts by affecting the turnover of various substrate proteins (28). Part of the fine-tuned regulation of CRL activity is due to cycles of NEDD8 conjugation onto a conserved lysine residue at the carboxy terminal domains of the corresponding cullins, and its removal. Neddylated cullins mark active CRLs and deneddylation of their cullins reduces their activity (28,29). Importantly, a large fraction of CRLs appears to be physically associated with CSN. CSN regulates CRL activity both by deneddylating the corresponding cullins and by sterically affecting their interaction with the corresponding E2 enzymes on one hand, and with their substrates on the other (29–31). Cullin neddylation also interferes with the association of the CSN-bound CRL to the protein CAND1, which regulates substrate receptor exchange (29,32–34). Thus, CSN's constant fine-tuning of the architecture and activity of CRLs in numerous aspects of cellular life make it a critical player in the same circuits. Resolution of CSN's structure has recently shown that it lays inactive until it encounters its substrates—neddylated cullins—which render it active (24).

CSN was previously shown to be involved in nucleotide excision repair—a DNA repair pathway which specifically handles bulky DNA lesions such as those caused by UV radiation (35). This involvement is based on CSN-mediated regulation of CRL complexes that contain cullin 4 (CRL4), which take part in the initial recognition and subsequent repair of UV-induced pyrimidine dimers (30,35–40).

Here, we reveal that CSN is a novel player in the vast signaling network that is activated by DSBs. We demonstrate that CSN is required for timely DSB repair and regulates the critical choice between DSB repair pathways. Specifically, CSN is required for the processivity of DNA deep end-resection, an initial, key step in HRR. Thus, upon induction of DSBs, a portion of cellular CSN is recruited both physically and functionally to serve the DSB response. Furthermore, our data suggest that CSN's functional partner in the DSB response is CRL4. We also establish that CSN is an ATM target and that the ATM-mediated phosphorylation on Serine 410 of its subunit, CSN3, is critical for its function in the DDR. Remarkably, elimination of this phosphorylation alone is sufficient to cause an organismal and cellular phenotype in the mouse. Thus, our results suggest that CSN is a new key regulator of the DSB response.

## MATERIALS AND METHODS

### Cell lines

Human U2-OS, HEK-293, and CAL51 cells were cultured in DMEM with 10% fetal bovine serum at 37°C in 5% CO_2_ atmosphere. U2-OS cells stably expressing ectopic GFP-CSN3 were grown in the same medium supplemented with 0.5 mg/ml G418. MEFs were isolated from mouse embryos on day E12.5–13.5 and cultured in HAM F-10 medium with 20% fetal bovine serum at 37°C in 3% O_2_ and 5% CO_2_ atmosphere in culture dishes pre-coated with 0.1% gelatin (Sigma–Aldrich, St. Louis, MO, USA).

### Chemicals, antibodies and immunoblotting

DharmaFECT1 transfection reagent was obtained from Dharmacon (GE Dharmacon, Lafayette, CO, USA). BrdU and Neocarzinostatin (NCS) were obtained from Sigma–Aldrich (St. Louis, MO, USA). The ATM inhibitor, KU60019 (41) and the DNA-PK inhibitor, NU7441 (42) were obtained from Tcris Bioscience (Bristol, UK). Anti-53BP1 mouse monoclonal antibody was a generous gift from T. Halazonetis, anti-53BP1 polyclonal antibody was obtained from Novus Biologicals, LLC (Littleton CO, USA), anti-pS139-H2AX (γH2AX) polyclonal antibody and polyclonal antibodies against CSN subunits 1, 3 and 5 were obtained from Bethyl Laboratories, Inc. (Montgomery, TX, USA), anti-RNF168, anti-H2A, anti-pS139-H2AX monoclonal antibody and anti-BRCA1 antibodies were obtained from Merck Millipore (Darmstadt, Germany), anti-H4 was obtained from Abcam (Cambridge, MA, USA), anti-HSC70 monoclonal antibody was obtained from Santa Cruz Biotechnology Inc. (Santa Cruz, CA, USA), and anti-Cullin 1 antibody was obtained from Invitrogen Corporation (Camarillo, CA, USA). Anti-mouse and anti-rabbit IgG, Alexa 488/568/633 were purchased from Molecular Probes (Leiden, Netherlands), and HRP-conjugated anti-rabbit IgG and anti-mouse IgG were obtained from Jackson Immunoresearch Laboratories, Inc. (West Grove, PA, USA). Anti-Cullin 4A was produced in the Kurz laboratory. The phospho-specific antibody against pS410 of CSN3 was produced by Bethyl Laboratories, Inc. Immunoblotting was carried out as previously described (43).

### Immunoprecipitation

Cells expressing GFP-tagged proteins were lysed in HBSS (340 mM sucrose, 15 mM Tris–Cl pH 7.5, 15 mM NaCl, 60 mM KCl, 10 mM dithiothreitol) in the presence of protease and phosphatase inhibitors. After 10 min on ice the nuclear fraction was separated by centrifugation at 650 g for 3 min at 4°C. Nuclei were lysed in RIPA buffer (50 mM Tris, pH 7.5, 15 mM NaCl, 1% Igepal, 0.1% sodium dodecyl sulfate, 0.5% sodium deoxycholate) in the presence of protease and phosphatase inhibitors. GFP-trap reagent (ChromoTek GmbH Martinsried, Germany) was used to immunoprecipitate the GFP-tagged protein. The immune complexes were subsequently subjected to immunoblotting analysis. Immunoprecipitation of endogenous proteins from whole cell extracts was carried out using RIPA buffer to lyse the cells and was followed by five consecutive washes with this buffer.

### RNA Interference

OnTarget Plus SMARTpool siRNAs targeting CSN subunits were obtained from Dharmacon: CSN3 (L-01149400), CSN1 (L-01227200) and CUL4A (L-012610). CSN5 siRNA was custom designed by Integrated DNA Technologies (IDT, Coralville, IA, USA) in order to target the following sequence: AGA AGU ACU UUA CCU GAA A. U2-OS cells were cultured to 30–50% confluence and transfected with siRNA using DharmaFECT1 following manufacturer instructions.

### Clonogenic survival assay

CAL51-derived cells and MEFs were plated in triplicate at densities of 100–3000 cells per 60 mm plate and incubated for 24 h before exposure to various doses of NCS. After 2 weeks in culture, cell colonies were fixed and stained with 0.2% crystal violet in 50% ethanol. Colonies containing at least 50 cells were counted under a dissection microscope.

### Immunostaining

Detection of 53BP1, γH2AX and Rad51, nuclear foci by immunofluorescence staining was performed as described previously (43). Nuclear foci were quantified using the ImageJ software (http://imagej.nih.gov/ij/). 200–300 cells were counted per time point.

### DSB repair pathways assay

The NHEJ/HRR balance assay was carried out as previously described (44), using U2-OS cells harboring a single copy of the SeeSaw 2.0 Reporter. Briefly, 5000 cells were plated on a 96-well plate. The cells were transfected with siRNAs using RNAiMAX (Life Technologies, Grand Island, NY, USA), and 2 days later were infected with a lentiviral vector expressing BFP-tagged I-SceI (45), at multiplicity of infection 5, and fresh medium was added 24 h later. Forty-eight hours later, the cells were fixed with 4% paraformaldehyde and washed with phosphate buffered saline (PBS) prior to scoring under a fluorescent microscope for blue, green and red fluorescence. NHEJ/HRR balance was calculated according to the ratio of green versus red cells, and this ratio was normalized against a control (irrelevant) siRNA. Statistical significance was determined using the paired sample *t* test.

### DNA end-resection

SMART (Single Molecule Analysis of Resection Tracks) was performed as previously described (46). Briefly, U2-OS cells transfected with either siRNA against CSN1 or a control siRNA were cultured in the presence of 10 μM bromodeoxyuridine (BrdU) for 24 h, irradiated with 10 Gy of IR and harvested 1 h later. Cells were embedded in low-melting agarose and in-gel DNA extraction followed. To stretch the DNA fibers, silanized coverslips (Genomic Vision, Bagneux, France) were dipped into the DNA solution for 15 min and pulled out at constant speed (250 μm/s). Coverslips were baked for 2 h at 60°C and incubated without denaturation with an anti-BrdU mouse monoclonal antibody. After washing with PBS, coverslips were incubated with a secondary antibody. The coverslips were mounted with ProLong^®^ Gold Antifade Reagent (Molecular Probes) and stored at −20°C. DNA fibers were observed using a Nikon NI-E microscope under a PLAN FLOUR40×/0.75 PHL DLL objective. The images were recorded and processed using NIS ELEMENTS Nikon software. For each experiment, at least 200 DNA fibers were analyzed, and the length of DNA fibers was measured using Adobe Photoshop CS4 Extended version 11.0 (Adobe Systems Incorporated). Statistical significance in these experiments was determined with the paired Student's *t*-test using the PRISM software (Graphpad Software Inc.).

### Induction of localized DNA damage

In order to detect recruitment of ectopic, GFP-tagged proteins to laser-induced DNA damage in live cells, U2-OS cells were plated on glass bottom dishes (MatTek) and pre-sensitized with 5 μM BrdU for 48 h at 37°C. The dishes were transferred into a microscope top-stage incubator equipped with a control system for gas mixture and humidity (Okolab, Ottaviani, Italy). DNA damage was induced on a Leica TCS SP8 confocal microscope (Leica Microsystems, Wetzlar, Germany) using a 405 nm Diode laser focused through an HC PL APO 63×, 1.4-numerical aperture oil immersion objective (8% laser power, scan speed 650 ms, 40 scans). Images were acquired using the same system. In order to detect recruitment of endogenous proteins to laser-induced sites of DNA damage by immunostaining, two-photon based micro-irradiation of DNA was performed using a focused 800 nm laser beam in LSM 510 Meta confocal microscope (Carl Zeiss, Jena, Germany) equipped with a Spectral-Physics Mai-Tai (Deep-See) multi-photon laser system focused through a 63× 1.25NA oil immersion objective (8.5% laser power, scan speed 256 ms, 40 scans at zoom ×1). Imaging of the immunostained cells was subsequently carried out using a Leica TCS SP5 confocal microscope.

### Generation of the Csn3 S410A knock-in mouse

Targeting vectors were generated in *Escherichia coli* via the Recombineering technique using BAC DNA. A bacterial artificial chromosome (BAC) clone spanning the Cops3 genomic locus (bMQ 302C20) was obtained from the 129S7/AB2.2 BAC clone library (the Sanger Institute), and served for subcloning. Further reagents and protocols were obtained from the National Institutes of Health, NCI-Frederick (see http://recombineering.ncifcrf.gov for details). Homologous recombination was carried out in SW102 bacteria as previously described (47). In short, a point mutation leading to the S410A substitution (TCA→GCA) was first introduced into the BAC clone using gal K selection. The targeting cassette was subsequently retrieved via gap repair into the pBS-derived vector, pL253. Insertion of a floxed neo cassette (red and green box) followed. For each retrieval or insertion step, mini targeting arms of 250–500 bp containing the preferred restriction sites were prepared using PCR. The targeting construct was linearized and introduced into 129/Sv male ES cells, which were subsequently doubly selected in medium containing 200 μg/ml G418 and 0.2 μM fialuridine. Positive clones were analyzed by Southern blotting using 5′ and 3′ probes (Supplementary Figure S7A) to distinguish between the mutant and wild-type alleles. Targeted ES cells were microinjected into 129/Sv blastocysts. Embryos were transferred to pseudopregnant foster mothers. Founder animals were identified by Southern blotting analysis of tail DNA using the 5′ and 3′ probes. Further genotyping was carried out using PCR (Supplementary Figure S7B).

## RESULTS

### CSN is an ATM target in response to induction of DNA damage

**Figure 1.**
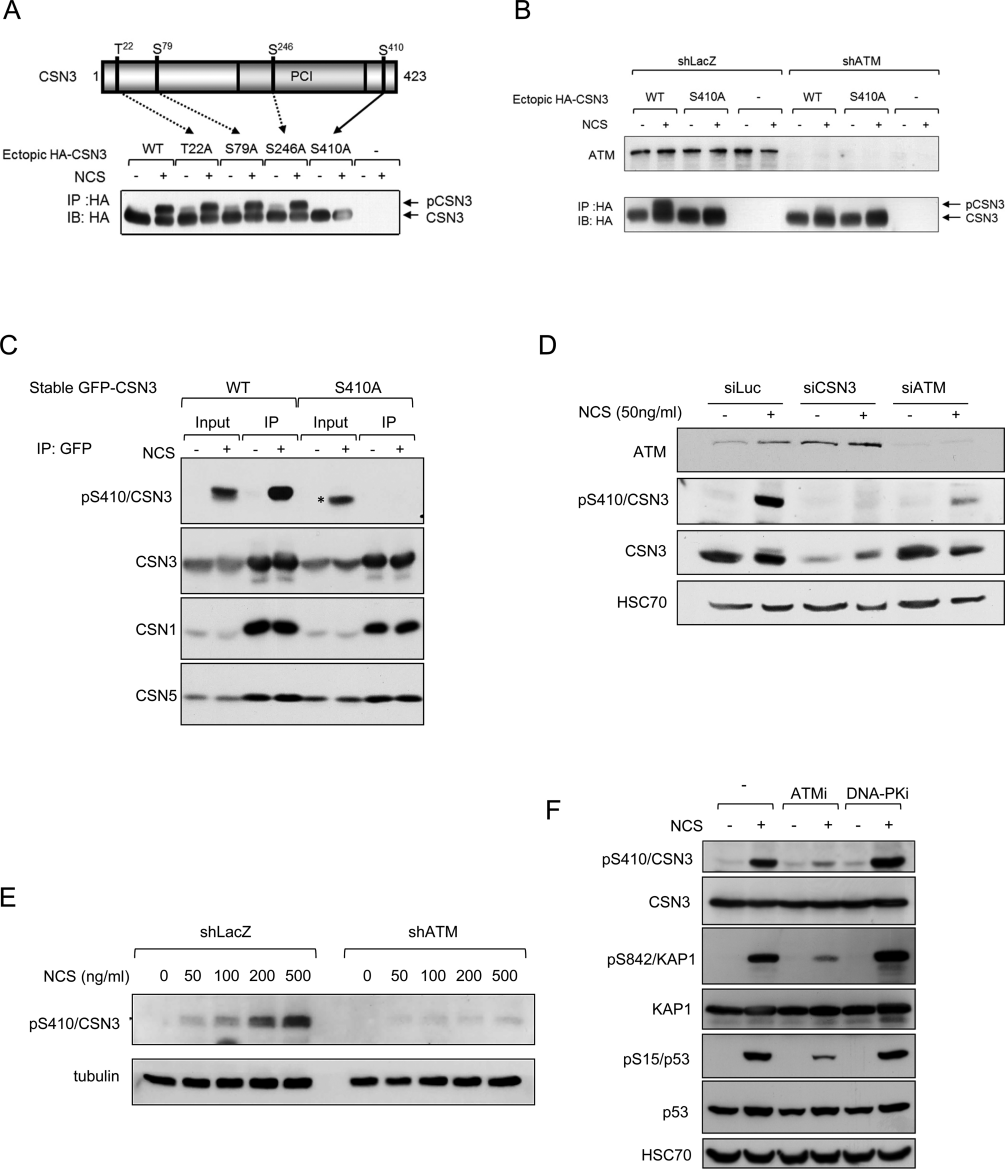

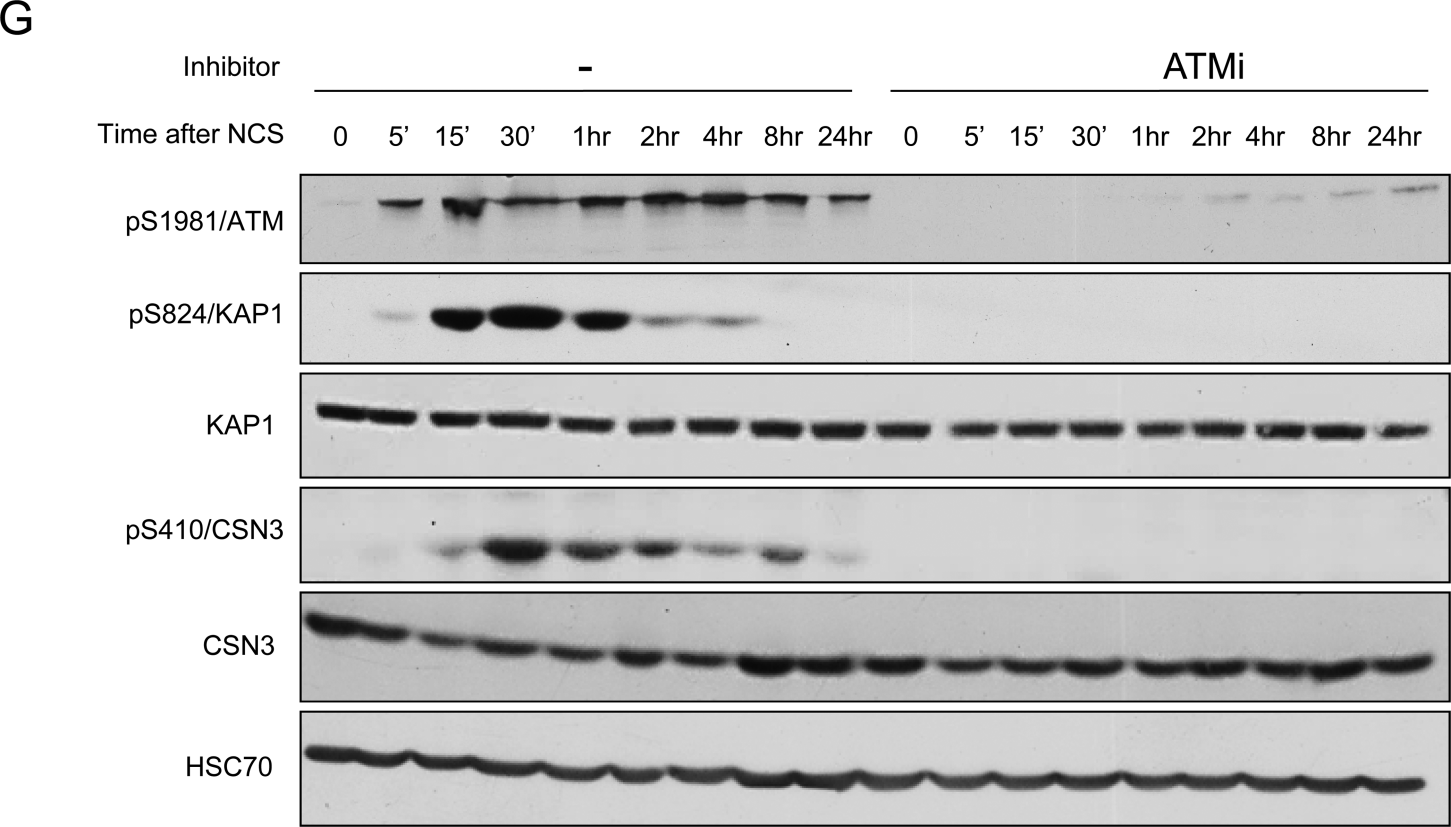
CSN3 is an ATM substrate in the DNA damage response. (**A**) Mapping of the phosphorylation site on CSN3. Potential ATM targets in this protein (serine or threonine residues followed by glutamine) were mutated in recombinant HA-tagged CSN3, and the various versions of the protein were expressed in HEK293 cells. The band-shift observed after DNA damage induction (500 ng/ml of NCS for 30 min) was specifically abolished by the S410A substitution, indicating that the corresponding modification occurred on S410. (**B**) Phosphorylation of S410 of CSN3 in response to DNA damage induction is ATM-dependent. The experiment described in (A) was repeated in HEK293 cells that stably expressed shRNA against ATM. Note the considerable reduction in CSN3's band-shift in cells depleted of ATM. (**C**) Detection of CSN3 phosphorylation in cells using a specific anti-phospho antibody. GFP-tagged CSN3 in wild-type and mutant (S410A) versions was ectopically expressed in U2-OS cells. Following treatment with 50 ng/ml of NCS for 1 h, ectopic CSN3 was immunoprecipitated using an anti-GFP antibody, and the immune complexes were blotted with the indicated antibodies. The ectopic wild-type protein, but not the mutant, reacts with the antibody after induction of DNA damage. In cellular extracts, the anti-phospho antibody detects the phosphorylation of another protein in response to DNA damage (asterisk). Note that endogenous CSN1 and CSN5 are pulled down by ectopic CSN3. (**D**) Depletion of CSN3 or ATM markedly reduces the pS410-CSN3 phosphorylation signal. U2-OS cells transfected with the indicated siRNAs were treated 72 h later with 50 ng/ml of NCS for 30 min and processed for immunoblotting analysis. (**E**) CSN3 phosphorylation is dose- and ATM-dependent. HEK293 cells expressing irrelevant (LacZ) or ATM shRNA were treated for 1 h with various doses of NCS, followed by immunoblotting with the indicated antibodies. (**F**) CSN3 phosphorylation is not dependent on DNA-PK. U2-OS cells were treated with the indicated inhibitors 30 min prior to treatment with 50 ng/ml of NCS. Phosphorylation of the ATM substrate KAP-1 on S824 served to monitor ATM activity in response to DNA damage induction. ATMi: the ATM inhibitor, KU60019 (41), applied at 5 μM concentration. DNA-PKi: the DNA-PK inhibitor, NU7441 (42) applied at 10 μM concentration. (**G**) Time course of phosphorylation of endogenous CSN3 following treatment of U2-OS cells with 50 ng/ml of NCS.

Early studies of the ATM protein in our lab included a search for ATM-interacting proteins using the two-hybrid assay. A bait spanning ATM residues 1184–1583, which contain a leucine zipper—a protein-protein interaction motif—identified CSN subunit 8 (CSN8) as prey (Supplementary Figure S1A). Co-immunoprecipitation of endogenous ATM and CSN8 supported the notion of a physical interaction between them (Supplementary Figure S1B), raising the possibility of functional interaction between ATM and CSN and possibly rendering CSN an ATM target. In order to search for DNA damage-induced phosphorylation of CSN subunits in cells, we expressed these subunits in HEK293 cells as ectopic HA-tagged proteins, and treated the cells with the radiomimetic drug neocarzinostatin (NCS) concurrently with a protein phospho-labeling pulse. This experiment revealed marked enhancement of phospho-labeling of CSN subunit 3 (CSN3) following NCS treatment (Supplementary Figure S1C). We further noticed that in response to DNA damage induction, a portion of CSN3 exhibited altered electrophoretic migration (‘gel shift’) (Figure 1A and B). Since this band-shift was largely abolished upon knockdown of ATM (Figure 1B), we assumed that it represented ATM-mediated phosphorylation of CSN3 in cells. In order to map the phosphorylation site, we expressed mutant versions of CSN3 in cells. In each mutant, one of its four S/TQ sequences—potential ATM target sites—was abolished by Ser→Ala substitutions. Only the S410A substitution eliminated the band-shift (Figure 1A), suggesting that the presumed phosphorylation occurred on Ser410. A polyclonal phospho-specific antibody raised to detect this assumed phosphorylation reacted strongly with ectopic wild-type CSN3 expressed following NCS treatment, but not with an S410A mutant version of this protein (Figure 1C). This result indicated that phosphorylation of CSN3 on Ser410 occurred in cells in response to DNA damage and was detected by the antibody. The antibody also detected the phosphorylation of endogenous CSN3, which was ATM- and dose-dependent (Figure 1D–G), and DNA-PK independent (Figure 1F); it peaked within 30 min of damage induction and subsided several hours later—a time course typical of many ATM-mediated protein phosphorylations (Figure 1G). These results established that Ser410 of CSN3 is an ATM target in response to DSB induction and suggested a role for CSN in the ATM-mediated DSB response.

### CSN is required for regulation of DSB repair

**Figure 2.**
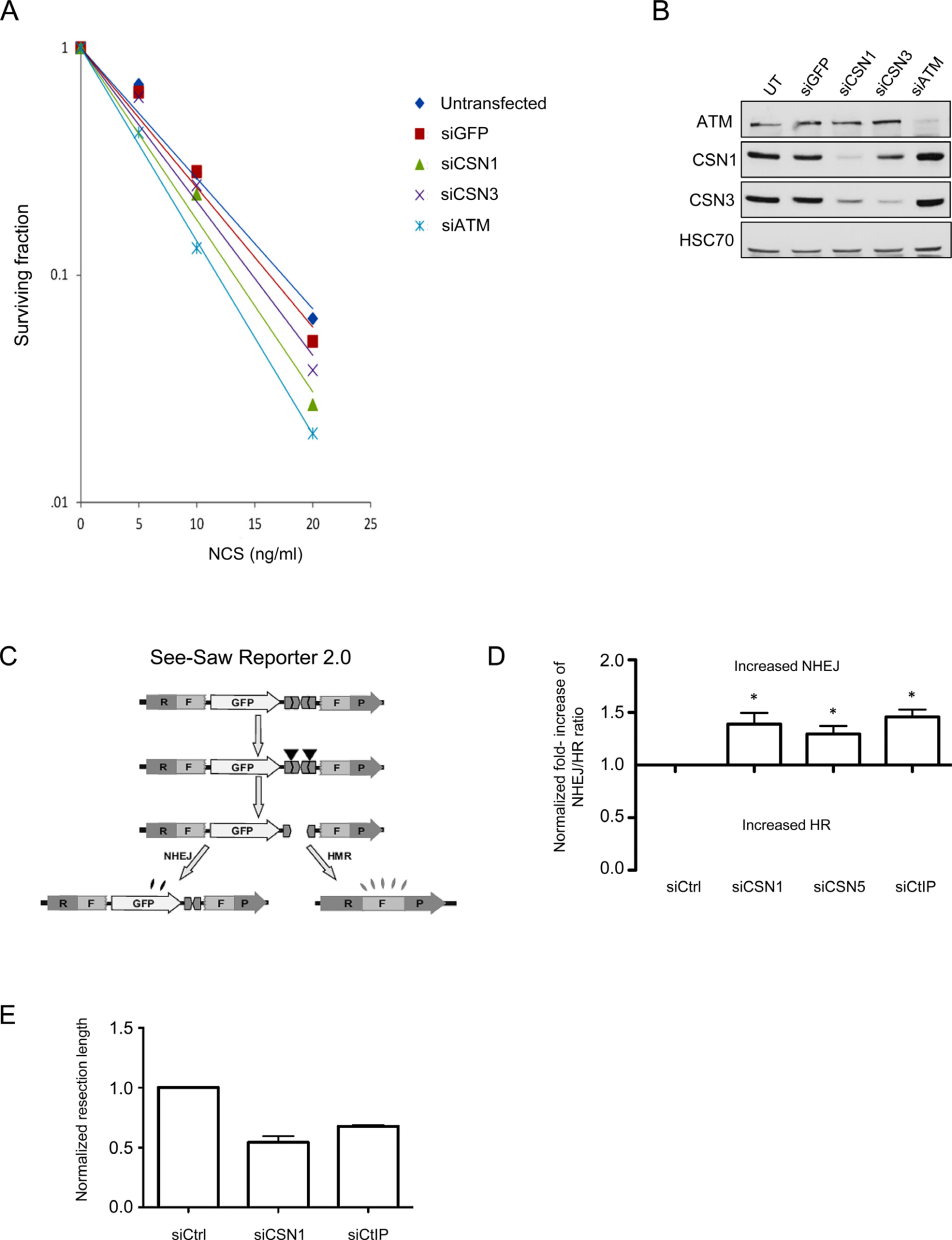
Depletion of CSN subunits affects the DNA damage response. (**A**) Clonogenic survival assay of CAL51 cells transfected with siCSN1 or siCSN3 for 72 h. The cells were treated with various concentrations of NCS. Cells transfected with siATM or siGFP served as controls. (**B**) Extent of protein depletion in the experiment depicted in (A). Note the reduction in CSN3 amounts in cells deleted of CSN1. (**C**) Schematic representation of the SeeSaw 2.0 reporters (44). A GFP ORF is flanked by two truncated parts of an RFP ORF (RF and FP) sharing 302 bp of homologous sequence. Two I-SceI-target sites in opposite orientation are present at the 3′ end of the GFP ORF. After generation of a DSB by ectopic I-SceI endonuclease, it may be repaired via NHEJ, and the cells will retain GFP expression, or it may be repaired via HRR, creating a functional RFP ORF. (**D**) Effect of the indicated siRNAs in the SSR 2.0 assay. Two CSN subunits were knocked down: CSN1, whose depletion leads to reduction of other CSN subunits (Supplementary Figure S2), and CSN5, which harbors the catalytic site of CSN's deneddylase activity. The ratio of green to red cells in each condition was calculated and normalized for each siRNA against the effect of a scrambled siRNA. Increased NHEJ/HRR ratio over the baseline value of 1.0 represents an imbalance between the two DSB repair pathways toward NHEJ. The data represent four sets of triplicate experiments. Paired *t* test was used for statistical analysis. Asterisk indicates a *P* value of <0.05. (**E**) Single Molecule Analysis of Resection Tracks (SMART) analysis of cells depleted of CSN1 or the end-resection regulator, CtIP, and control cells. The length of individual fibers was measured as described in the ‘Materials and Methods’ section and the median of at least 250 fibers was calculated. The average of the medians and the standard deviation of three independent experiments are shown. Note the marked reduction in resection extent of CSN1-depleted cells compared to CtIP depletion (siControl-siCSN1: *P* = 0.0133; siControl-siCtIP: *P* = 0.0003).

We further examined the role of CSN in the DSB response by measuring the cellular sensitivity of cells depleted of CSN subunits CSN1 or CSN3, to the cytotoxic effect of NCS, using RNAi. Deficiency of DDR players that disrupts DSB repair or interferes with the fine balance between DSB repair pathways usually results in cellular hypersensitivity to DSB-inducing agents. This defective phenotype is typically measured by clonogenic survival assays. Since CSN functions as a holoenzyme (24), loss of specific subunits is expected to compromise its integrity and activity. We observed that depletion of CSN1 led to concomitant depletion of two other CSN subunits, CSN3 and CSN5, as did CSN3 depletion with regard to CSN1 and CSN5 (Supplementary Figure S2). Knockdown of the catalytic subunit, CSN5, did not lead to either CSN1 or CSN3 depletion (Supplementary Figure S2), but presumably rendered CSN inactive. Depletion of either CSN1 or CSN3 increased cellular sensitivity to radiomimetic treatment (Figure 2A and B), suggesting that down-regulation of CSN affected DSB repair.

Several assays have been developed in order to assess individually the efficiency of the two main pathways of DSB repair, NHEJ and HRR, in cultured cell lines. A recently established assay measures the ratio between these two pathways (44). Abrogation of either repair pathway disturbs the finely regulated equilibrium between the two mechanisms, such that deficiencies in DDR players that abrogate HRR increase the NHEJ/HRR ratio, and vice versa. Deviation from the normal ratio between NHEJ and HRR can lead to defective DSB repair (44,48–50). To determine this ratio, we used U2-OS cells whose genome contains the SeeSaw Reporter (44) (SSR 2.0; Figure 2C), which harbors a cleavage site of the meganuclease I-SceI, that normally does not cleave human DNA. Following I-SceI-mediated cleavage in these cells, repair of the break by NHEJ leads to green GFP fluorescence whereas repair via HRR leads to red RFP fluorescence, and the ratio between the two is determined. The reporter is constructed such that direct, error-free ligation of the sticky ends formed by I-SceI is prevented. Figure 2D shows that down-regulation of either CSN1 or the catalytic subunit, CSN5, in these cells increased the NHEJ/HRR ratio similarly to the effect of CtIP down-regulation. CtIP is an important regulator of the NHEJ:HRR balance as it regulates DNA end-resection—an essential step toward HRR (3,51). This effect is comparable to that obtained by the depletion of several other DDR players, such as those that mobilize major ubiquitin-driven DDR pathways (44). Importantly, this effect was not due to abrogation of cell cycle dynamics as a result of CSN depletion, as this depletion did not affect the cell cycle distribution (not shown).

HRR reduction could be attributed to reduced efficiency of a key step in this repair pathway—deep 5′ to 3′ resection at each DSB ends. This step promotes HRR while inhibiting NHEJ and is therefore highly regulated (52,53). In order to assess the effect of CSN depletion on end-resection processivity we used SMART (Single Molecule Analysis of Resection Tracks)—a recently developed, high-resolution assay that measures the extent of end-resection directly at the DNA level (54). Importantly, CSN1 depletion significantly reduced the length of resected DNA in IR-treated cells, to a greater extent than that observed in cells depleted of CtIP, a major regulator of this process (Figure 2E). We thus inferred that CSN is a critical regulator of proper HRR by influencing end-resection, thereby affecting the maintenance of the delicate balance between the major DSB repair pathways.

### CSN is recruited to sites of DNA damage

**Figure 3.**
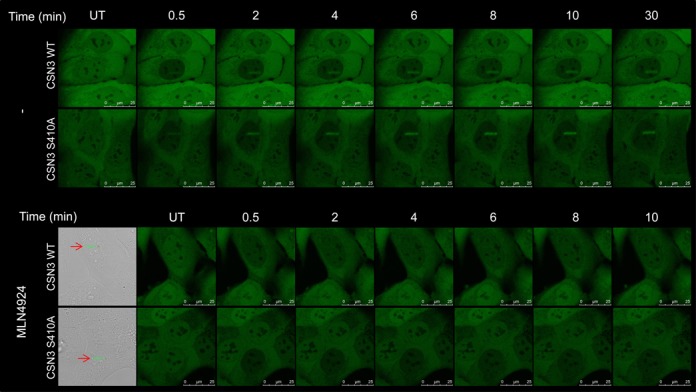
Recruitment of ectopic, GFP-tagged CSN3 to sites of laser-induced DNA damage. U2-OS cells stably expressing ectopic, GFP-tagged CSN3 in wild-type or mutant (S410A) versions were either treated or not with 3 μM of the neddylation inhibitor, MLN4924, for 6 h. Laser micro-irradiation was then carried out and images captured in live cells at the indicated time points. Experiments were carried out in at least 15 cells. Representative cells are shown. In the bottom panel, red arrows and green lines indicate the location of laser-induced damage in bright-field images of the irradiated cells.

The above data strongly suggested that CSN participates in processes that occur at DSB sites. A common characteristic of proteins that function at these sites is their temporary recruitment to areas of chromatin spanning DSBs (4). Often, only a portion of the cellular content of the protein is recruited, but even the relocalization of such a small portion can be demonstrated experimentally by targeting a narrow nuclear sector with a focused laser beam to induce localized, dense DNA damage, and subsequently imaging the protein recruitment to that sector. Using this system, we were able to observe recruitment of ectopic, stably expressed GFP-tagged CSN3 to such ‘laser stripes’ as early as 30 s after induction of DNA damage, culminating within several minutes (Figure 3). This recruited fraction was retained at the damaged sites for several hours (not shown). Since immunoprecipitation of ectopic CSN3 concomitantly pulls down endogenous CSN1 and CSN5 (Figure 1C), we assume that the recombinant protein is incorporated into the CSN complex, and therefore its recruitment to DSB sites represents the relocalization of the CSN holoenzyme to these sites.

The recruitment dynamics were largely similar for both the wild-type and the S410A-mutant proteins (Figure 3). Furthermore, the recruitment of wild-type CSN3 was not affected by the addition of an ATM inhibitor, suggesting that the ATM-mediated phosphorylation of CSN3 did not play a major role in this process. Importantly, however, pre-treatment of the cells with the small molecule, MLN4924—a selective inhibitor of the NEDD8-activating enzyme (NAE) that initiates the neddylation process (55)—abolished the recruitment of CSN3 but not of two other DDR players expressed as ectopic proteins: polynucleotide kinase phosphatase (PNKP) (Supplementary Figure S3), and the E3 ubiquitin ligase, RNF8 (not shown). This is a striking situation, in which the recruitment of a single DDR player to sites of DNA damage is differentially inhibited by a small molecule.

These results indicate that CSN belongs to the growing group of proteins that are summoned from their ordinary duties in other physiologic arenas to function in the DDR. In this case, CSN's involvement in the DDR is dependent on prior protein neddylation, suggesting that its role in the DDR might be associated with its activity as a deneddylase. Depletion of CSN1, which leads to depletion of other CSN subunits (Supplementary Figure S2), did not affect the recruitment of several major players in the early stage of the DSB response—specifically 53BP1, RNF168 and BRCA1 (Supplementary Figure S4)—indicating that CSN is not required for their recruitment to the damaged sites. CSN may therefore participate in a stage of the DSB response which is crucial for well-timed DSB repair, but not in the initial build-up of the protein hubs surrounding DSB sites.

### Cullin 4A partners with CSN in the DSB response

**Figure 4.**
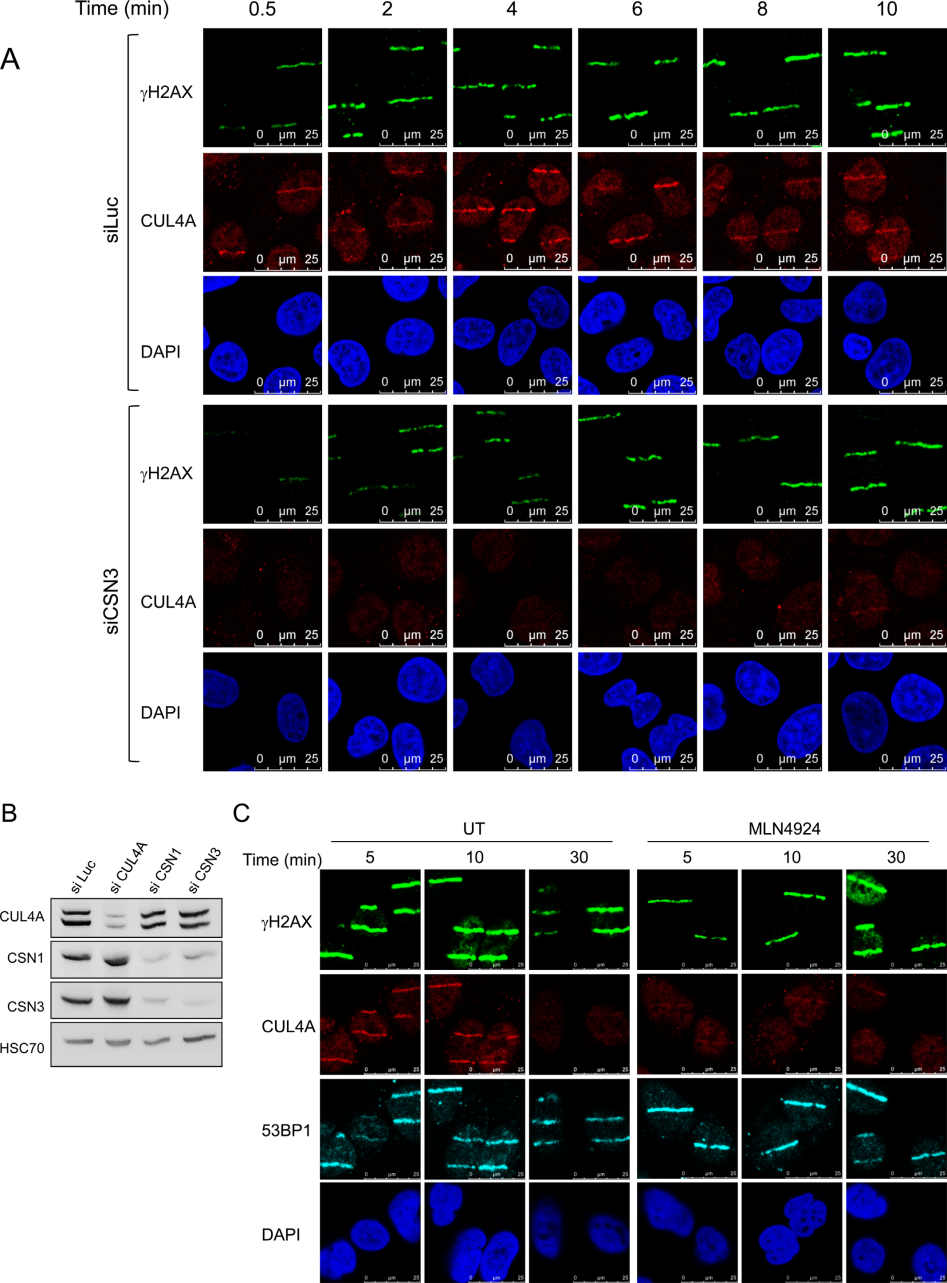
Recruitment of cullin 4A to sites of laser-induced DNA damage is CSN- and neddylation-dependent. (**A**) Localized DNA damage was induced using a focused laser microbeam in U2-OS cells transfected with the indicated siRNAs. At various time points after DNA damage induction the cells were processed for immunostaining with the indicated antibodies. (**B**) Extent of knockdown of different proteins in this experiment demonstrated using immunoblotting. Note that down-regulation of CSN subunits does not affect CUL4A levels, and vice versa. (**C**) Similar experiment as in (A), with treatment of the cells with 3 μM of the neddylation inhibitor, MLN4924, for 6 h prior to damage induction.

Among the CRLs, CRL4 (which contains cullins 4A or 4B) has been reported to be involved in a variety of DNA transactions, such as transcription, replication and repair (56) and has been specifically shown to play a central role in the sensing and repairing of bulky DNA lesions caused by UV radiation, via the nucleotide excision repair (NER) pathway (30,35–38,56–57). We asked whether a functional link between CSN and CUL4A ubiquitin ligase exists in the context of the DSB response. We used an antibody specific for human CUL4A (Supplementary Figure S5) to follow the recruitment of endogenous CUL4A to laser stripes induced in human U2-OS cells. Endogenous CUL4A was rapidly recruited to sites of DNA damage (Figure 4A), and gradually dissipated over the next 30 min (Figure 4C). Notably, recruitment of CUL4A depended on the presence of both CSN3 (Figure 4A) and CSN1 (Supplementary Figure S6A), suggesting that this process requires a functional CSN. Moreover, pre-treatment of the cells with the neddylation inhibitor, MLN4924, markedly attenuated CUL4A's recruitment to sites of DNA damage (Figure 4C), without affecting the recruitment of two other major DDR players - 53BP1 (Figure 4C) and RNF168 (not shown). These results suggest that CUL4A accrual at sites of DNA damage requires active NEDD8 turnover, since it is impaired by inhibiting either protein neddylation or cullin deneddylation. Crucially, ATM inhibition did not interfere with CUL4A recruitment (Supplementary Figure S6B), indicating that, as with CSN recruitment, this process did not require phosphorylation of ATM targets.

### ATM-mediated phosphorylation of CSN3 is required for timely DSB repair

Most DDR players are recruited from other processes and typically undergo PTMs that modulate their function or activity in order to prepare them for their temporary tasks in the DDR (6,8); ATM-dependent phosphorylation is a paramount example for such a PTM in the DSB response (11). The results above suggested that ATM-mediated phosphorylation of CSN3 may not play a critical role in its physical recruitment to sites of DNA damage. Is this phosphorylation necessary for CSN's function in the DDR? Our approach to studying the functional significance of this phosphorylation was based on abolishing the phosphorylation site in murine Csn3 by knocking-in the corresponding mouse gene, *Cops3* such that it will produce a protein with an S410A substitution. An animal with a non-phosphorylatable Csn3 exhibiting an organismal and cellular phenotype would be valuable for demonstrating the importance of this phosphorylation. The sequences of the human and murine ortholog proteins share 99% identity, and the amino acid sequence spanning Ser410 is identical in the two proteins. The *Cops3* gene was thus targeted to induce the appropriate amino acid substitution in its protein product (Supplementary Figure S7).

**Figure 5.**
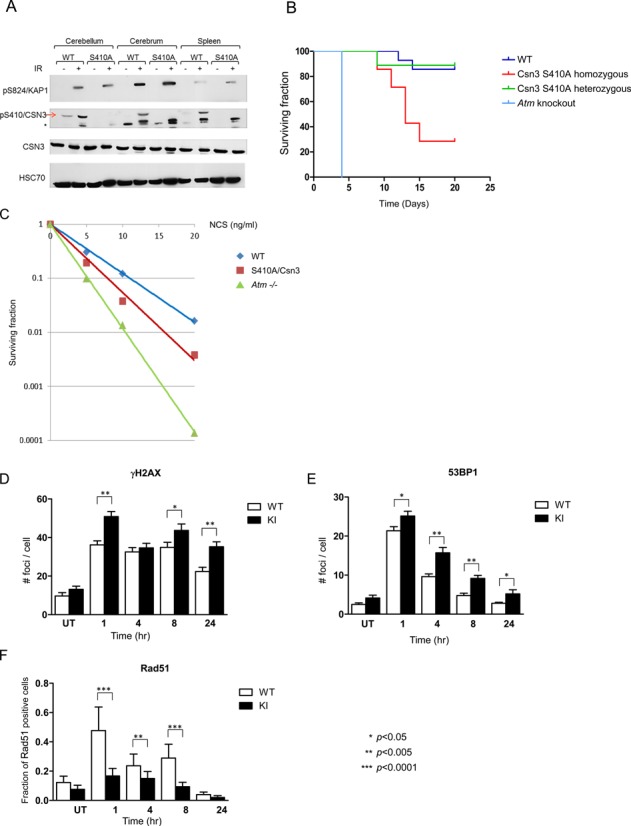
Elimination of the Atm phosphorylation site on murine endogenous Csn3 impairs the DSB response in mice. (**A**) Endogenous Csn3 is not phosphorylated on S410 in response to DSBs in various tissues of mice expressing a mutated protein with the S410A amino acid substitution. Ten-day-old mice were irradiated with 10 Gy of IR, and 1 h later tissues were collected and processed for immunoblotting analysis with the indicated antibodies. Phosphorylation of the Atm substrate Kap-1 on S824 served to monitor ATM activity. (**B**) Kaplan–Meier survival curve of 1-month old mice with various genotypes in the Cops3 locus encoding Csn3 following whole body irradiation with 6.5 Gy of IR. 10–20 animals of each genotype were irradiated. (**C**) Clonogenic survival of MEFs with different genotypes after treatment with various NCS doses. Cells expressing non-phosphorylatable Csn3 exhibit intermediate radiomimetic sensitivity between that of wild-type and that of Atm-deficient cells. (**D** and **E**). Lack of an Atm phosphorylation site on Csn3 slows the disappearance of DNA damage-induced γH2AX and 53BP1 nuclear foci. MEFs with the indicated version of endogenous Csn3 were irradiated with 1 Gy of IR, and subsequently fixed and stained with antibodies against γH2AX or 53BP1. Nuclear foci were counted in 150–250 cells per time point. (**F**) Similar analysis as in (D) and (E) using an antibody against Rad51. The diagram presents the fraction of cells which contained more than 15 nuclear foci. Experiments were carried out in triplicate, and one set of results is shown. Error bars represent SEM. Statistical analysis was based on Student's *t* test for γH2AX, 53BP1 and *χ*2 for Rad51.

Mice homozygous for the mutant *Cops3* allele expressed normal levels of Csn3 (Figure 4A). As expected, following whole body X-irradiation, Csn3 was not phosphorylated in tissues of these animals, contrary to another Atm substrate, Kap-1 (Figure 5A). These mice grow normally, have a normal life span and do not exhibit excessive morbidity, suggesting that the S410A amino acid substitution in Csn3 does not considerably affect CSN function under normal conditions. Significantly, however, these mice exhibited increased radiosensitivity that was intermediate between that of wild-type and *Atm*−/− knockout mice (Figure 5B). Moreover, mouse embryo fibroblasts (MEFs) with non-phosphorylatable Csn3 showed NCS sensitivity that was intermediate between that of wild-type and Atm-deficient MEFs (Figure 5C), similar to human cells with down-regulated CSN subunits (Figure 2A). In order to monitor DSB repair in these cells, we followed the dynamics of two hallmarks of unrepaired DSBs in MEFs treated with ionizing radiation: nuclear foci of phosphorylated histone H2AX (γH2AX) (58), and nuclear foci of the DDR player 53BP1 (59). The gradual disappearance of both of these DSB features was retarded in MEFs expressing the mutant Csn3 (Figure 5D and E), attesting to a perturbation of DSB repair in these cells. The results therefore indicate that CSN's involvement in DSB repair relies on the ATM-mediated phosphorylation of CSN3, although this phosphorylation does not seem to play a role in the physical recruitment of CSN to the sites of DNA damage.

In view of the result suggesting reduced HRR in human cells depleted of CSN subunits (Figure 2D), we applied an immunofluorescent assay to the MEFs in order to assess successful initiation of HRR. We quantified the nuclear foci of a major HRR player, Rad51, which coats DNA single strand stretches following deep end-resection. Remarkably, at various time points following IR treatment, the fraction of cells with Rad51 nuclear foci in the mutant MEFs was significantly lower than in wild-type cells (Figure 5F), attesting to a reduction in deep end-resection at DSB sites in the mutant MEFs. The organismal and cellular phenotypes associated with the loss of just one of the numerous ATM-mediated phosphorylations (11) are notable and suggest that this phosphorylation plays a crucial role in the DDR, specifically in determining DSB repair efficiency.

## DISCUSSION

Our results establish CSN as a new, crucial player in the cellular response to DSBs, and place it at a critical decision point in DSB repair, together with CRL4. Furthermore, we show that the loss of a single ATM-mediated phosphorylation on one CSN subunit is sufficient to affect the organismal and cellular response to DSBs.

The DDR network is based on a highly efficient and economic system which temporarily borrows functional modules from other processes and adapts them to the needs of the DDR, often by inducing PTMs on their key targets (1–2,11). The dynamic organization of CRLs that endows them with a special capacity to alter their substrate specificity (28) makes them ideal candidates for such a scenario. Our data indicate that CSN and CRL4 are temporarily adapted and recruited to serve the DSB response. Abrogation of CRL4 recruitment in CSN-depleted cells as well as the similar inhibition of CSN and CUL4A recruitment by the neddylation inhibitor, MLN4924, suggests that CSN and CRL4 are bound together in the same process that requires protein neddylation dynamics.

Our data also demonstrate a role for this process in the regulation of a key step in HRR—DNA end-resection—which appears to be under very tight regulation (52,53) and subsequently, affects the ratio between HRR and NHEJ. There is growing appreciation for the role of this critical balance in making DSB repair a well-timed and smooth process, and, accordingly, of its subjection to several regulatory mechanisms (9,44). Presumably, the involvement of CSN and CRL4 in this process is due to ubiquitylation of CRL substrate(s) at the sites of DNA damage.

In the nucleotide excision repair pathway, which repairs bulky DNA adducts, recognition of the DNA lesion by the substrate receptor, DDB2, triggers the dissociation of CSN from the CRL4^DDB2^ complex, thereby enhancing its activity, which is subsequently directed to NER players and core histones at the damaged sites (30,38). It was recently shown that inositol hexakiphosphate kinase-1 (IP6K1) is involved in UV damage-induced dissociation of CSN from CRL4 (40). Interestingly, IP6K1 was previously shown to be required for the HRR branch of DSB repair (60). In our study, we found that in the DSB response, CUL4A and CSN are recruited to the damaged sites with roughly similar kinetics. Analogous to the NER scenario, CRL4 and CSN might be initially complexed, with their subsequent disengagement leading to modulation of CRL4 activity and substrate receptor exchange. It will now be important to identify the substrate receptor(s) engaged by the recruited CRL4 out of the 50 or so that characterize CRL4s (56), to examine whether CSN is involved in regulation of their exchange at DSB sites, and to identify the relevant downstream CRL4 substrates.

ATM-mediated phosphorylation is an important PTM in the recruitment of functional modules from other processes to DDR service. ATM phosphorylates a vast number of downstream substrates in the DDR (11–13). Our results with knock-in mice expressing the mutant S410A Csn3 and with cells derived from them, demonstrate that an important phosphorylation can be singled out of this noisy background if it has high functional significance. It is interesting that this single phosphorylation plays a crucial role in accumulation of RAD51 at sites of DSBs, suggesting that it directly impacts the deep end-resection which is a pre-requisite for HRR. Notably, putative phosphorylation of Ser410 of CSN3 was noticed in two proteomic screens that studied phosphoproteome dynamics in response to IR (12) or to the topoisomerase II inhibitor, etoposide, a DSB inducer (61). Our data, together with the visibility of this phosphorylation in proteomic screens, mark it as a highly significant event in the DSB response in general. Recent mass-spectrometric analysis of CSN subunits after treatment of cells with UV radiation suggested that several sites are phosphorylated on CSN in that context too, but these sites were not further validated and their functional significance was not examined (62). It will not be surprising, however, if this modification shapes CSN's action in the nucleotide excision repair pathway as well.

Our data do not point to Ser410 phosphorylation as important for CSN recruitment to sites of DNA damage; thus, its functional significance should be sought in the possible modulation of CSN's activity through highly dynamic engagement with its neddylated cullin substrates (29,63). ATM typically phosphorylates more than one protein in a pathway and even within the same protein complex (11). Indeed, in the phosphoproteomic screen for ATM/ATR targets carried out by Matsuoka*et al*. (12), Ser232 of CSN subunit 7A was a hit, in addition to Ser410 of CSN3. Importantly, both serine residues are located in the carboxy-termini of the corresponding CSN subunits. The recent elucidation of CSN structure by Lingaraju *et al*. (24) revealed that the carboxy termini of all CSN subunits, which typically contain α-helices, protrude out of the main body of the CSN particle and together form a helical bundle. Thus, both ATM-mediated phosphorylations of CSN fall within this helical bundle. Lingaraju *et al*. (24) suggested that the helical bundle enables the catalytic subunit, CSN5, to sense the assembly state of CSN and become activated in the presence of a CRL substrate. We assume, therefore, that together, these phosphorylations affect the substrate recognition of CSN in the microenvironment of the protein hubs that assemble at sites of DNA damage. CSN's multi-pronged regulation of CRL architecture (28) may further impact the substrate receptor exchange of the interacting CRL, and subsequently, the ubiquitylation of specific proteins by that CRL. Notably, in the CSN structure, CSN3, which is an ATM target, and CSN8, which anchors ATM to CSN, are positioned adjacent to each other and close to the substrate receptor in the CSN-bound CRL (24). Work is in progress to identify the substrate receptor in the fraction of the CRL4 complex that is recruited to serve the DDR, and the downstream substrate of this CRL4 fraction.

All this converges to meticulous regulation of CRL4 at DSB sites, within the protein conglomerates that span these lesions. Such fine regulation of protein ubiquitylation agrees with the growing notion that this PTM must be carefully fine-tuned in the DDR and is therefore subjected to delicate checks and balances (7). In this context, the fine interplay between CRLs and their major regulator, CSN, is emerging as an important pathway regulatory junction, safeguarding the complex system of genome stability.

## SUPPLEMENTARY DATA

Supplementary Data are available at NAR Online.

SUPPLEMENTARY DATA
