# Supplementary material for: The COP9 signalosome is vital for timely repair of DNA double-strand breaks
Source: Nucleic Acids Res. 2015 Apr 8;43(9):4517–30. doi: 10.1093/nar/gkv270 (PMC4482063; doi:10.1093/nar/gkv270)
Supplement: SUPPLEMENTARY DATA [file supp_43_9_4517__index.html]

The COP9 signalosome is vital for timely repair of DNA double-strand breaks — The COP9 signalosome is vital for timely repair of DNA double-strand breaks — SUPPLEMENTARY DATA 

# The COP9 signalosome is vital for timely repair of DNA double-strand breaks

## SUPPLEMENTARY DATA

**Files in this Data Supplement:**

- SUPPLEMENTARY DATA
- SUPPLEMENTARY DATA
