# Supplementary material for: The COP9 signalosome is vital for timely repair of DNA double-strand breaks
Source: Nucleic Acids Res. 2015 Apr 8;43(9):4517–30. doi: 10.1093/nar/gkv270 (PMC4482063; doi:10.1093/nar/gkv270)
Supplement: SUPPLEMENTARY DATA [file supp_gkv270_nar-00441-d-2015-File003.pdf]

M. Meir et al:

The COP9 signalosome is vital for timely repair of DNA double-strand breaks

Supplementary Material

Legends to Supplementary Figures

Figure S1. Functional interaction between CSN and ATM. A. Schematic diagram showing the ATM-derived bait and its prey in a 2-hybrid screen, in which CSN8 was a hit. The numbers indicate amino acid positions in the shown proteins. PI3K: the PI3-kinase-like domain that harbors ATM's catalytic site. The PCI (common to Proteasome, COP9 and eIF3) domain in CSN8 is common to CSN subunits 1-4, 7 and 8 and is essential for CSN assembly. **B.** Physical interaction between ATM or p53, and CSN8. CSN8 was immunoprecipitated from HEK293 cells, and the immune complexes underwent immunoblotting analysis with the indicated antibodies. NRS: normal rabbit serum. **C.** Phosphorylation of CSN subunits in response to DNA damage. HA-tagged CSN subunits were ectopically expressed in HEK293 cells. Following metabolic phospholabeling and NCS treatment (500 ng/ml, 30 min), ectopic proteins were immunoprecipitated using an anti-HA antibody, and the immune complexes were subjected to gel electrophoresis followed by autoradiography or immunoblotting with anti-HA. Shown are the analyses of CSN3, 5 and 6. Of all CSN subunits, CSN3 clearly exhibited hyperphosphorylation following DNA damage induction.

Figure S2. Effect of depletion of specific CSN subunits on the cellular level of others. U2-OS cells were transfected with the indicated siRNAs and analyzed by immunoblotting with the indicated antibodies. In these cells, depletion of CSN1 and CSN3 led to a reduction in the amount of the other two subunits examined in this experiment, but not depletion of CSN5 – the catalytic subunit of the complex.

Figure S3. Recruitment of ectopic PNKP to laser-induced DNA damage is not affected by inhibition of protein neddylation. U2-OS cells stably expressing GFP tagged PNKP were plated and sensitized with BrdU as in Figure 3, and were either treated or not with 3 μM of MLN4924 for 6 hr. Laser micro-irradiation and live-cell imaging were carried out as in Figure 3. Experiments were carried out in at least 15 cells. Representative cells are shown.

Figure S4. CSN depletion does not affect the recruitment of 53BP1, RNF168, and BRCA1 to laser-induced DNA damage. A-C. U2-OS cells were transfected with the indicated siRNAs and 72 hr later localized DNA damage was induced using a focused laser microbeam. The cells were fixed at various time points following damage induction and stained with antibodies against the indicated proteins. **D**. Extent of CSN1 knockdown in this experiment demonstrated using immunoblotting analysis.

Figure S5. Specificity of the anti-CUL4A antibody in immunostaining assay. U2-OS cells were transfected with the indicated siRNAs for 72 hr and underwent immunostaining with an anti-CUL4A antibody. Note the marked reduction in the staining

signal upon CUL4A depletion. Immunoblotting analysis demonstrating the extent of CUL4A knockdown in this experiment is shown in Figure 4B.

Figure S6. CSN1 depletion but not ATM inhibition affects the recruitment of CUL4A to sites of laser-induced DNA damage. U2-OS cells were transfected with the indicated siRNAs for 72 hr (A) or treated with 5 µM of the ATM inhibitor, KU60019 (ATMi), for 1 hr (B), prior to induction of localized DNA damage. The cells were fixed at various time points following damage induction and stained with the indicated antibodies.

Figure S7. Gene targeting strategy for generating a CSN3/S410A knock-in mouse. **A.** We used the recombineering method, which is based on homologous recombination in E. Coli (see http://recombineering.ncifcrf.gov) to generate the targeting vector (upper panel). The vector was derived from a bacterial artificial chromosome spanning the murine *Cops3* gene, which encodes Csn3. A point mutation leading to the amino acid substitution, S410A, was introduced into the BAC, and the targeting cassette was retrieved via gap repair into pBS-derived vector, pL253 (yellow boxes). Insertion of a floxed Neo cassette (red and green box) followed. The pink boxes represent mini targeting arms used for retrieving the targeting cassette from the BAC. The targeting vector served to introduce the mutation into the genome of ES cells. Distinction between wild-type and mutant alleles (lower panels) was made using Southern blotting analysis (not shown). Dashed lines denote the bands expected to be detected by 5' and 3' probes prepared using PCR. Exon 12* contains the mutation leading to the S410 amino acid

substitution (TCA→GCA). Three primers were used for PCR-based genotyping (red arrows 1-3). **B.** PCR analysis demonstrating the WT, knock-in heterozygous and knock-in homozygous genotypes. The 723 bp fragment (obtained with primers 1+3, Panel A) represents the wild-type allele. The mutant allele yields the 810 bp fragment when primers 2+3 are used. Note that a 2 kb fragment defined by primers 1+3 on the mutant allele template was not formed under these PCR conditions.
