# Supplementary figures and images for: The COP9 signalosome is vital for timely repair of DNA double-strand breaks
Source: Nucleic Acids Res. 2015 Apr 8;43(9):4517–30. doi: 10.1093/nar/gkv270 (PMC4482063; doi:10.1093/nar/gkv270)

Figure S1

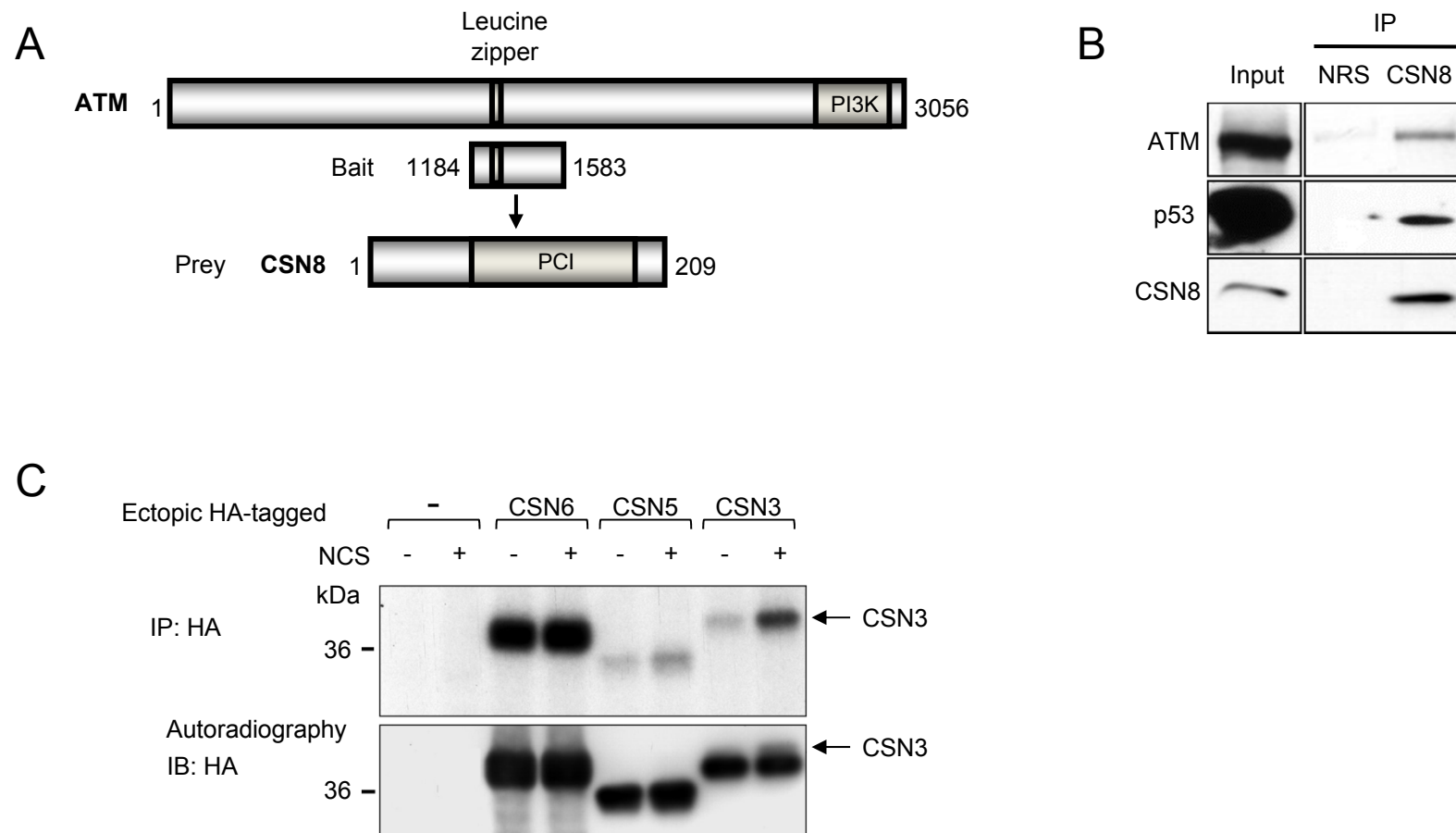

Figure S2

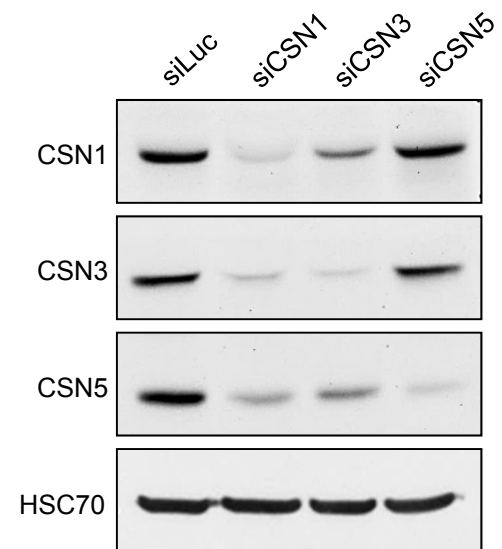

Figure S3

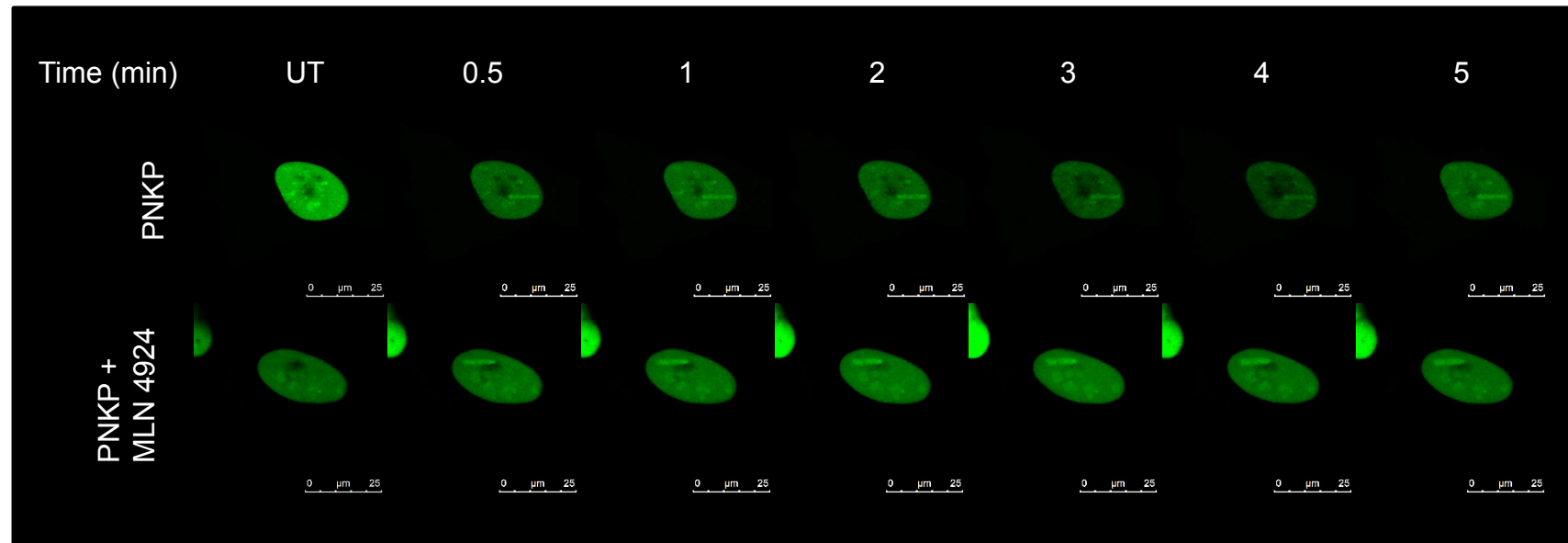

Figure S4

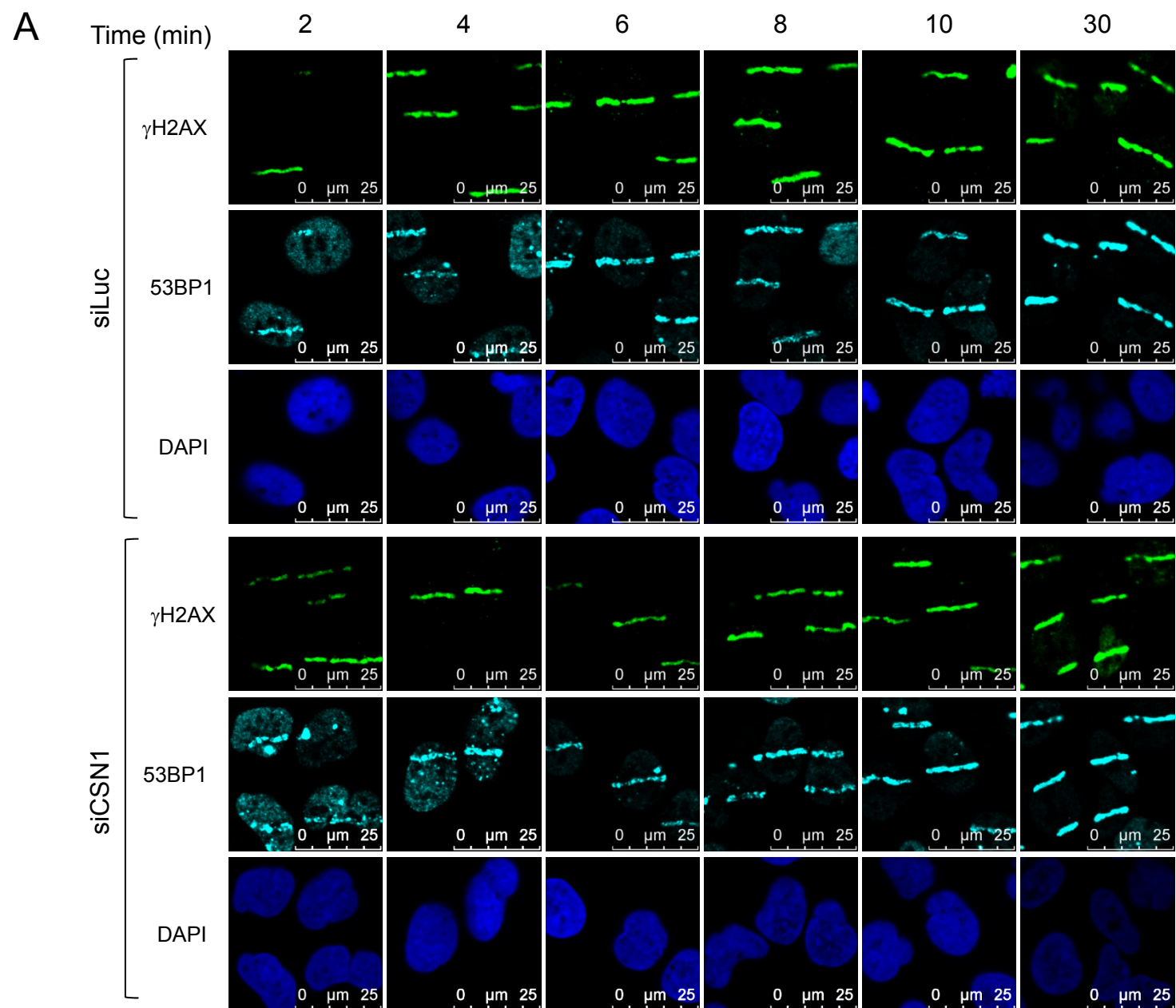

Figure S4

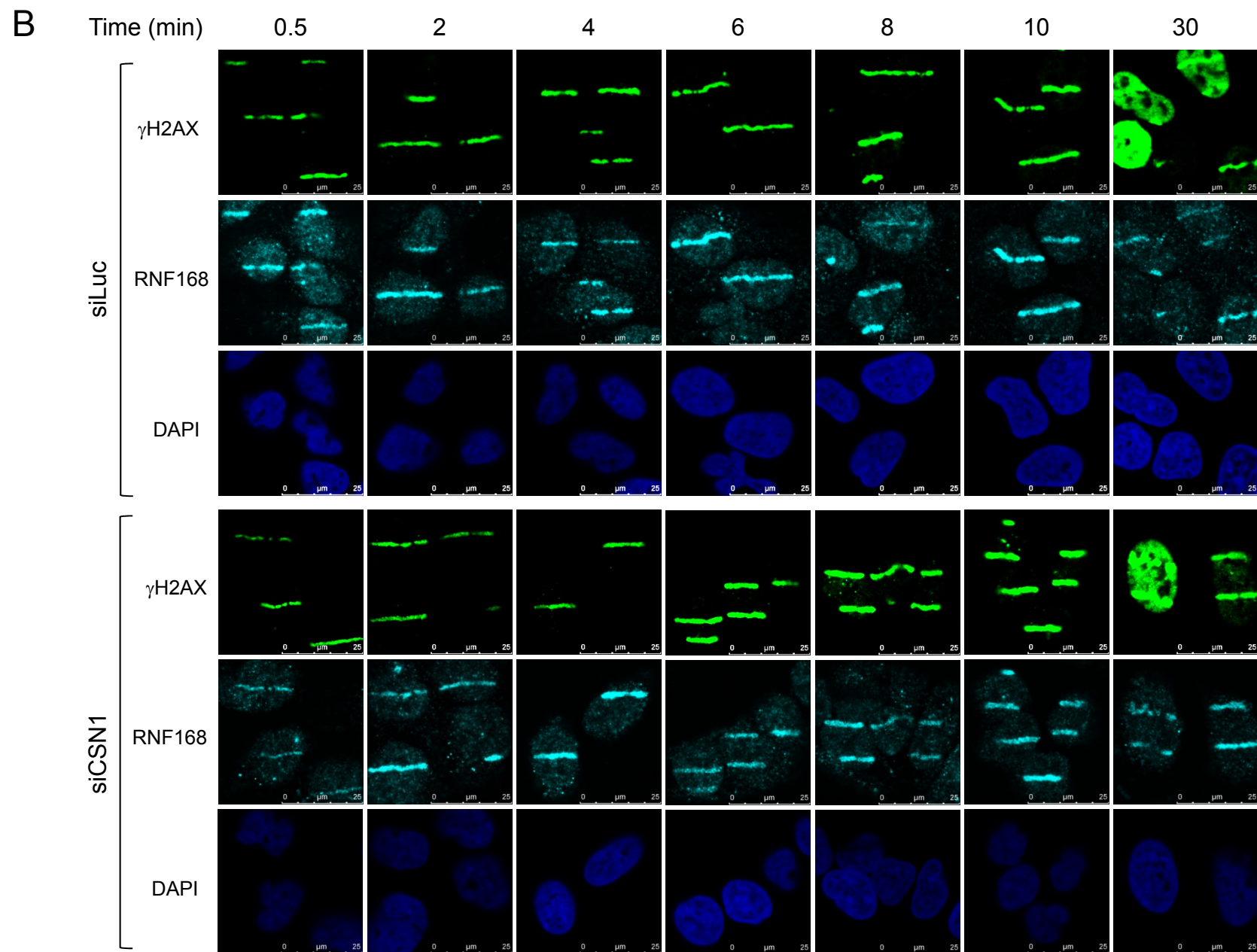

Figure S4

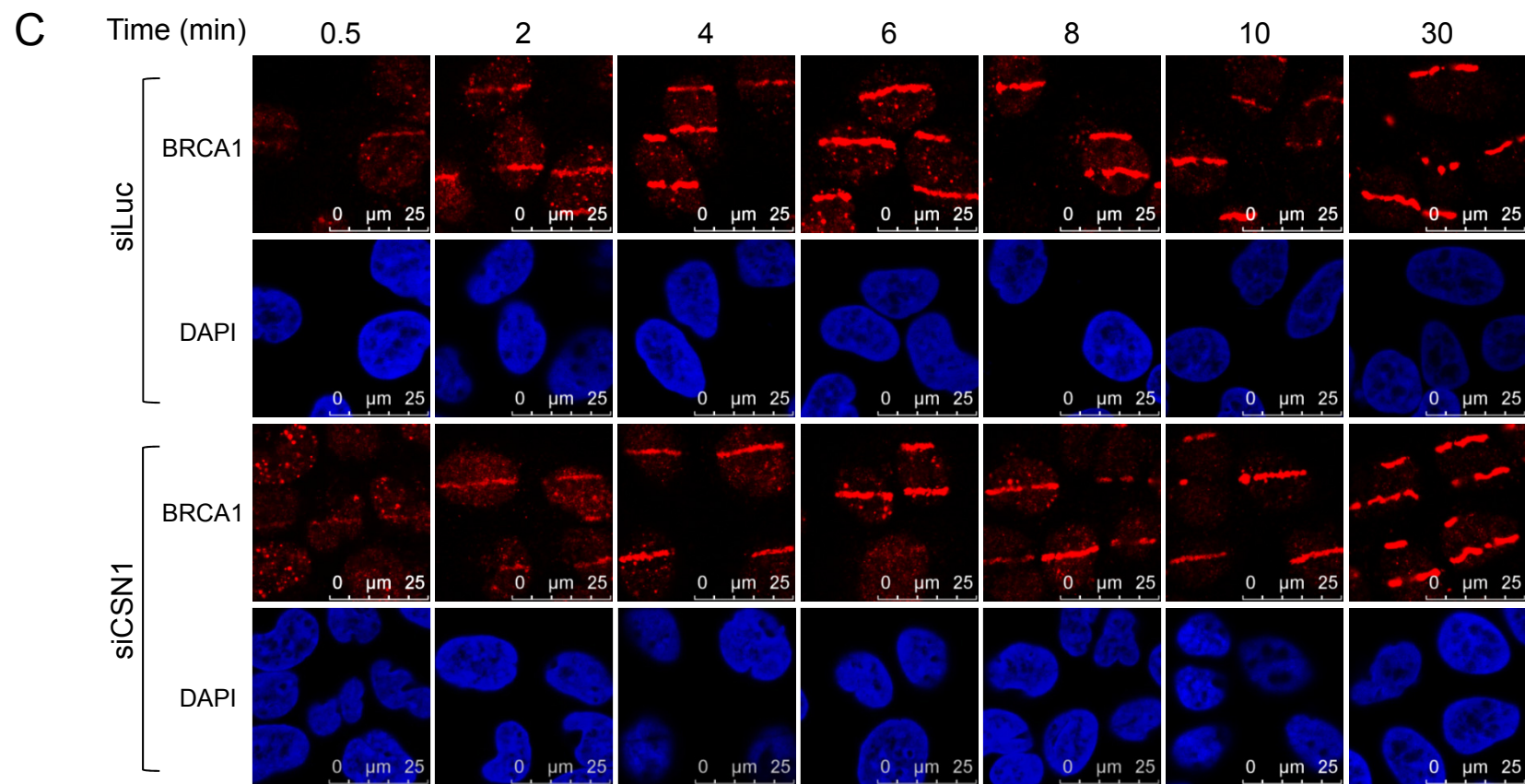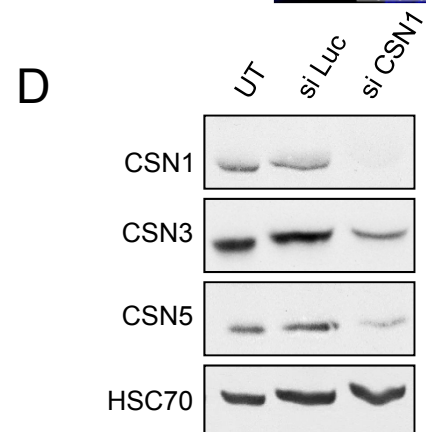

Figure S5

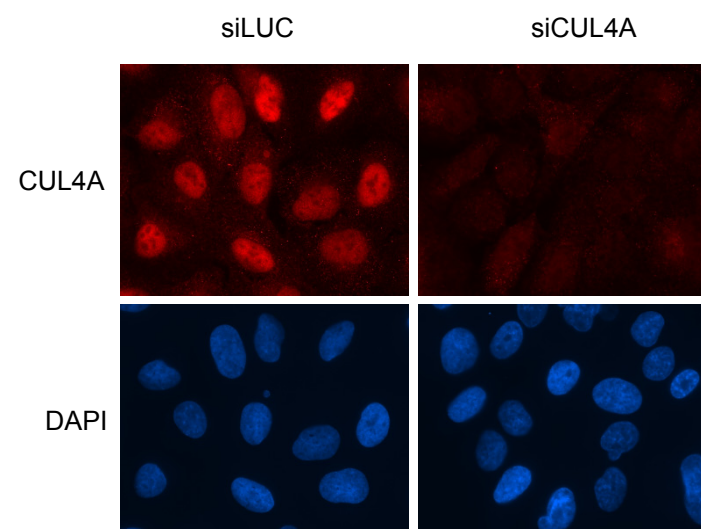

## Figure S6

A

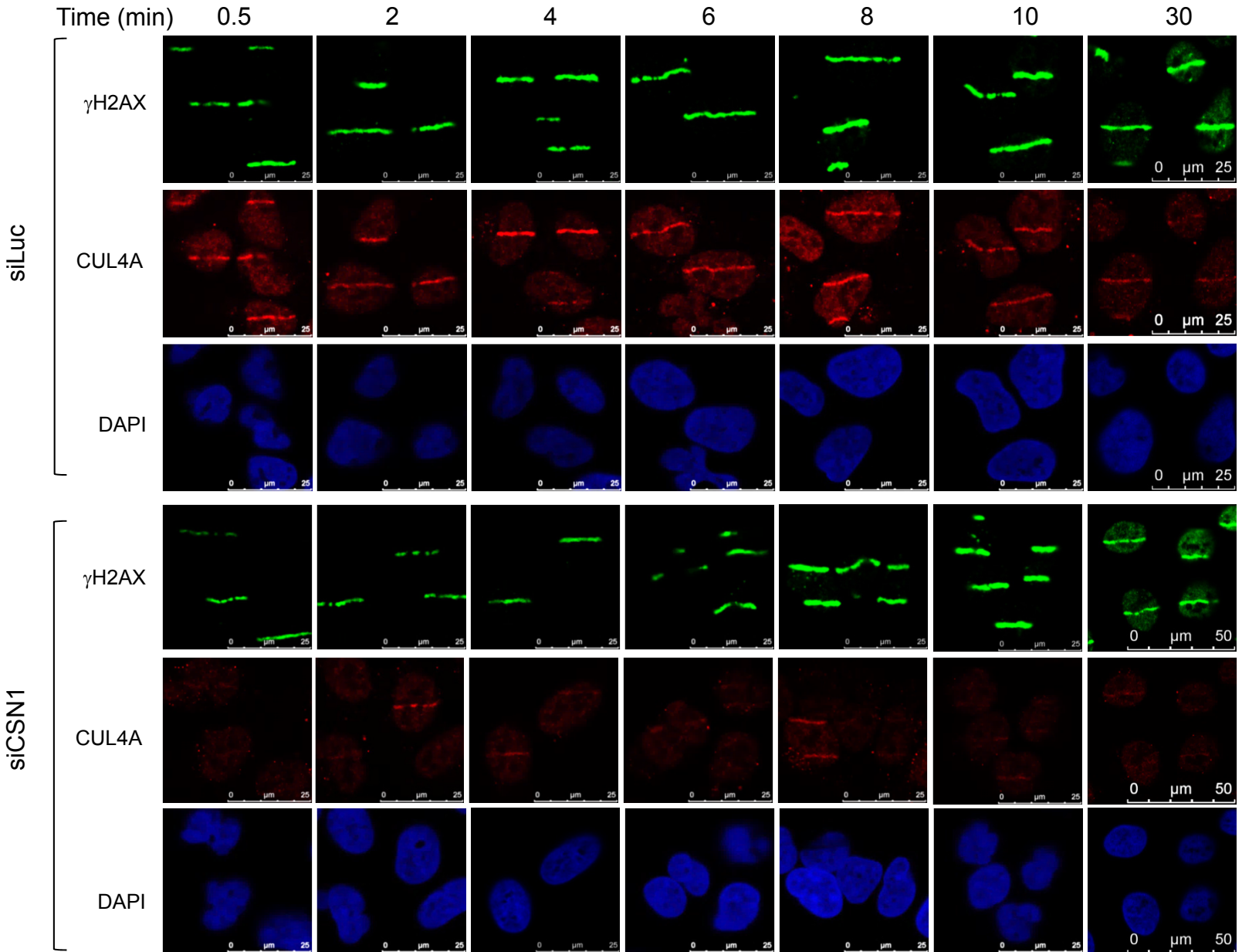

Figure S6

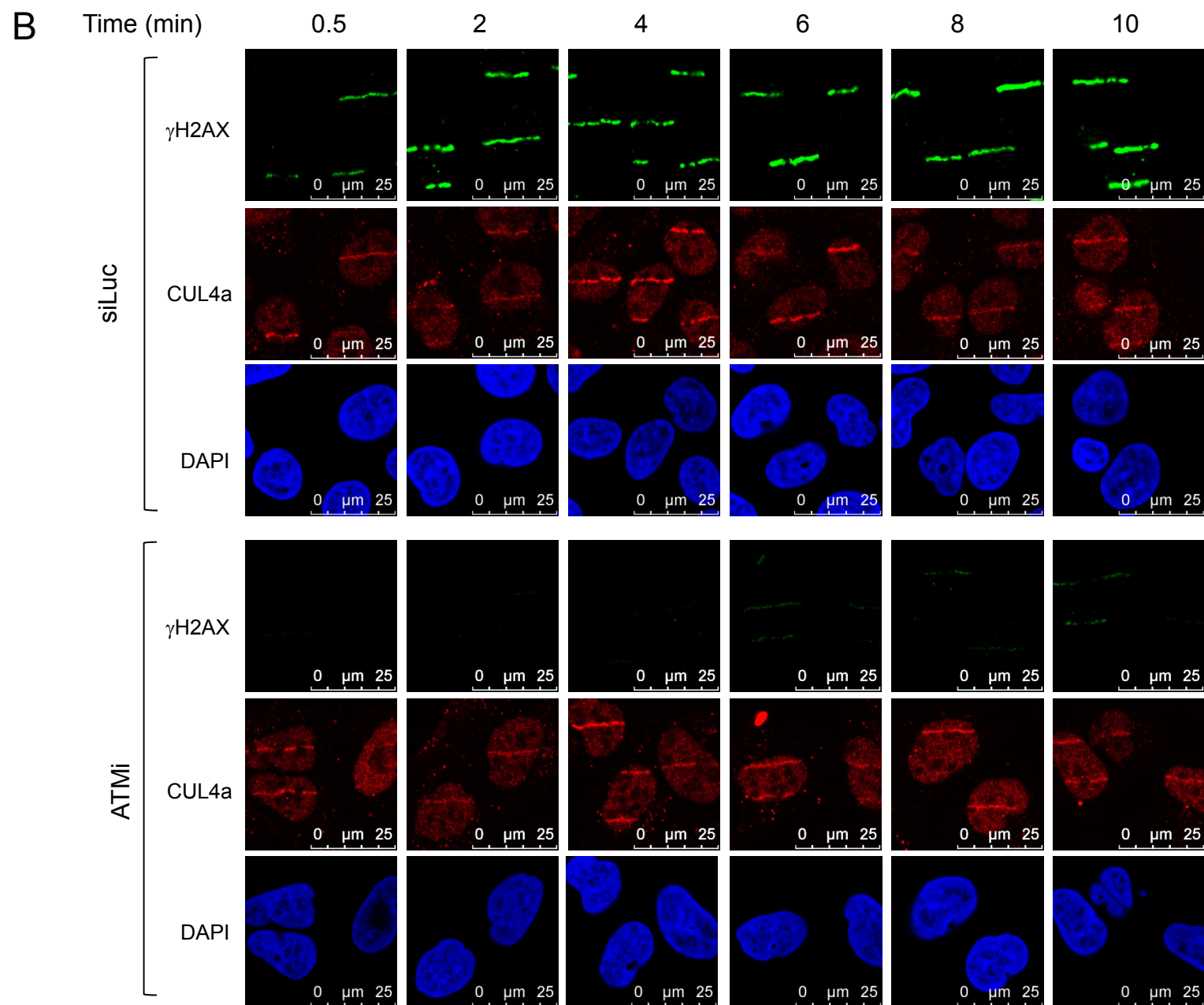

Figure S7

A

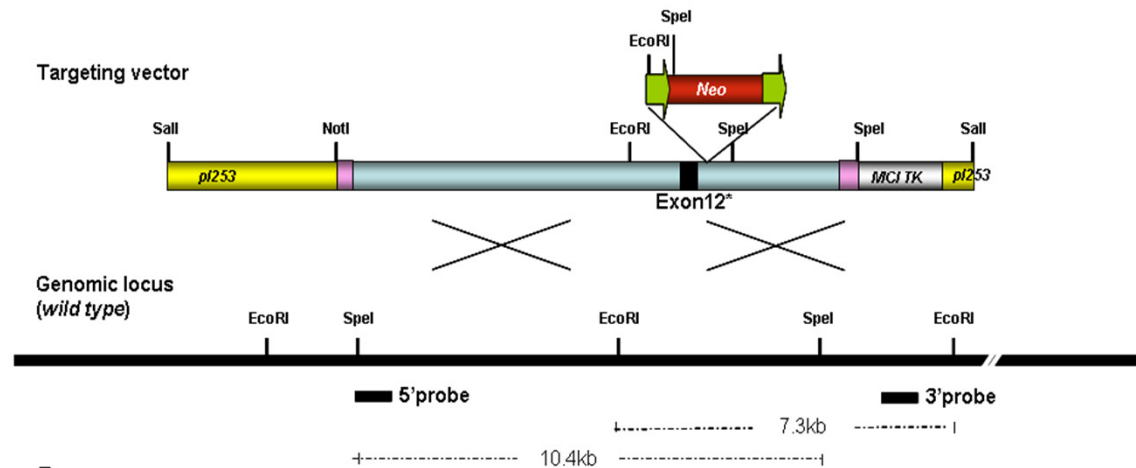

B.

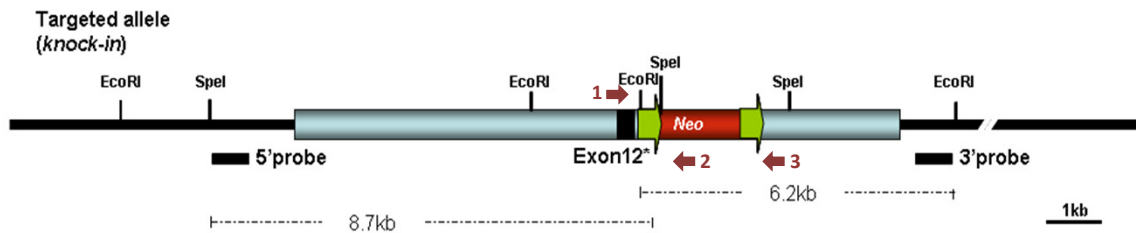

B

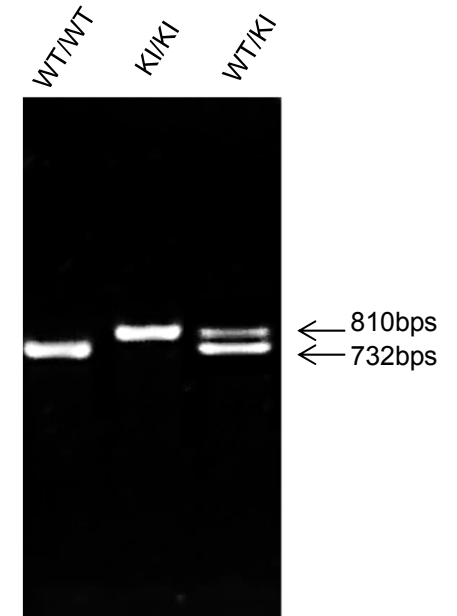

Supplement: SUPPLEMENTARY DATA [file supp_gkv270_nar-00441-d-2015-File004.pdf]
